# Evaluation of Corneal Endothelium in Adolescents with Juvenile Glaucoma

**DOI:** 10.1155/2015/895428

**Published:** 2015-01-06

**Authors:** Beata Urban, Alina Bakunowicz-Łazarczyk, Marta Michalczuk, Małgorzata Krętowska

**Affiliations:** ^1^Department of Pediatric Ophthalmology and Strabismus, Medical University of Bialystok, Waszyngtona 17, 15-274 Bialystok, Poland; ^2^Department of Pediatric Ophthalmology and Strabismus, Waszyngtona 17, 15-274 Bialystok, Poland; ^3^Bialystok University of Technology, Wiejska 45A, 15-351 Bialystok, Poland

## Abstract

*Purpose*. To evaluate the endothelial cell density (ECD) and central corneal thickness (CCT) in adolescents with juvenile open-angle glaucoma (JOAG) and ocular hypertension (OH) and to investigate the influence of topical antiglaucoma medications on ECD and CCT in adolescents with JOAG. *Methods*. ECD and CCT were investigated in 66 eyes of 33 adolescents with JOAG. Depending on the topical treatment the eyes were classified into 4 groups: (1) topical carbonic anhydrase inhibitor, (2) prostaglandin analogs, (3) beta-blocker, and (4) CAI-beta-blocker combination. ECD and CCT were also checked in 24 adolescents with OH and in control group (33 persons). *Results*. ECD was significantly lower in eyes with JOAG (2639.5 cells/mm^2^) compared with ECD in eyes with OH (2924.5 cells/mm^2^) and in control group (2955.5 cells/mm^2^). CCT was 0.554 mm in eyes with JOAG, 0.55 mm in eyes with OH, and 0.544 mm in control group. ECD in patients with JOAG was 2730 cells/mm^2^ (1 group), 2773.5 cells/mm^2^ (2 group), 2539.5 cells/mm^2^ (3 group), and 2551 cells/mm^2^ (4 group). CCT was 0.556 mm in 1 group, 0.558 mm in 2 group, 0.532 mm in 3 group, and 0.544 mm in 4 group. *Conclusions*. Our findings indicate that JOAG and OH did not affect CCT, but JOAG has influence on ECD in adolescents. There were no significant differences between ECD and CCT of eyes treated with different kinds of antiglaucoma medications.

## 1. Introduction

The corneal endothelium is a single layer of uniformly sized cells with hexagonal shape. Their amount decreases by approximately 100–200 cells per year [1]. The endothelial cell dysfunction is observed in myopia and contact lense wear in young patients [2, 3]. The decreasing number of endothelial cells can also be a result of diabetes mellitus [4]. Many studies have shown that even minor changes in the morphology of the endothelial cells may manifest in the disturbances in the tightness of the endothelial barrier. It has been demonstrated that human corneal endothelial cells have mitotic ability in vitro, but in vivo they are arrested in G1-phase of the cell cycle [5]. Loss of cells is compensated through the expanding and spreading of cells, which over time results in a corneal oedema. Prevention of the corneal endothelium dysfunction, its early detection, and immediate treatment is therefore crucial, especially if the problem concerns young patients. Corneal thickness and cell density are indirect measures of endothelial function, which are quickly obtained, reproducible, and reflective of clinically important direct toxic effects on the endothelium by the determination of any compromise of endothelial functional reserve [6]. Noncontact specular microscopy, which evaluates endothelial morphology quickly and easily, can be especially useful with children.

One of the conditions, which affect the cornea, is glaucoma. There are some reports concerning the analysis of the corneal endothelium in adults with different types of glaucoma; however, there are very few publications concerning the analysis of the cornea in juvenile patients with this disease [7–10]. Besides, topical glaucoma medications are widely used for childhood glaucoma, although little is known concerning the use of the newer glaucoma medications in this population. The aim of our study was to evaluate the endothelial cell density in patients with juvenile open-angle glaucoma (JOAG), treated with topical pressure-lowering medications and in young patients with untreated ocular hypertension (OH).

## 2. Materials and Methods

The current study was performed in the Department of Pediatric Ophthalmology and Strabismus, Medical University of Bialystok, Poland. This investigation received approval from University Ethic Committee and adhered to the tenets of the Declaration of Helsinki. For the purpose of this studywe retrospectively examined 66 eyes of 33 patients with JOAG (13 boys and 20 girls). The age of these patients was 11–17 years (mean: 15.57 ± 1.7 years). All patients were treated with topical pressure-lowering medications and the mean duration of this treatment was 2.927 ± 2.29 years and ranged from 6 months to 9 years. All patients were divided into four groups according to different classes of anti-glaucoma medications: (1) topical carbonic anhydrase inhibitors, Azopt, Alcon (CAI) (9 patients—18 eyes), (2) prostaglandin analogs, Xalatan, Pfizer (PGA) (11 patients—22 eyes), (3) beta-blocker, Betoptic, Alcon (BB) (10 patients—20 eyes), and (4) topical carbonic anhydrase inhibitors/beta-blocker combination, Cosopt, MSD (CAI-BB) (3 patients—6 eyes). No severe systemic or ocular adverse effects were observed in patients treated with topical antiglaucoma drugs. We also examined 24 patients (10 boys and 14 girls) aged from 11 to 17 years (mean: 13.54 ± 2.28 years) with untreated OH, with IOP between 24 and 30 mmHg, without any visual field defect or optic disk cupping. As controls, 66 eyes of 33 patients (18 boys and 15 girls) were examined. The mean age of the control group was 10–17 years (mean: 14.42 ± 2.32 years). Subjects were excluded if they had any of the following criteria: any changes in topical medications, history of any anterior segment disease, current use of contact lenses or discontinuation of contact lens use within 6 months of study entry, laser treatment, ocular trauma, and intraocular surgery. Examination of each subject consisted of measurement of best-corrected visual acuity, slit-lamp biomicroscopy, and measurement of IOP with Tono-Pen. The mean value of IOP was taken from nine measurements for each eye. A binocular indirect ophthalmoscopy fundus examination was also performed. The corneal endothelium in its central part and corneal thickness were diagnosed using a noncontact specular microscope Topcon SP-2000P. Several pictures were taken until a clear image of the endothelium in the central region was obtained. The endothelial cell count was performed using built-in image analysis software. On clear image 50 cells were counted manually. The image with the analyzed data was then printed out.

### 2.1. Statistical Analysis

For ECD and CCT the normal distribution hypothesis was discarded (the normality of distribution was examined using the Shapiro-Wilk test) and nonparametric tests were used: the Kruskal-Wallis test and multiple comparison tests. Differences with a *P* value less than 0.05 were considered statistically significant.

## 3. Results

**Figure 1:**
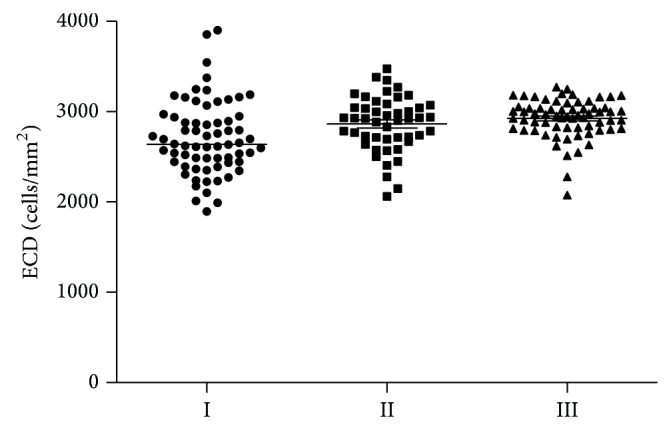
ECD values in patients with juvenile open-angle glaucoma (I group), ocular hypertension (II group), and the control group (III group).

A group of 33 adolescents with juvenile glaucoma treated with topical pressure-lowering medications were examined. All subjects had mean IOP < 21 mmHg in both eyes. The mean duration of antiglaucoma treatment was 2.93 ± 2.29 years. The median value for endothelial cell density ECD in patients with juvenile glaucoma (I group) was 2639.5 cells/mm^2^ and was significantly lower than in the control group (III group) (2955.5 cells/mm^2^) (*P* < 0.0001; multiple comparison test). The median value for endothelial cell density in patients with ocular hypertension (II group) was 2924.5 cells/mm^2^ and was not significantly lower than in the control group (*P* = 0.7; multiple comparison test). ECD values for the three examined groups are presented in Figure 1.

**Figure 2:**
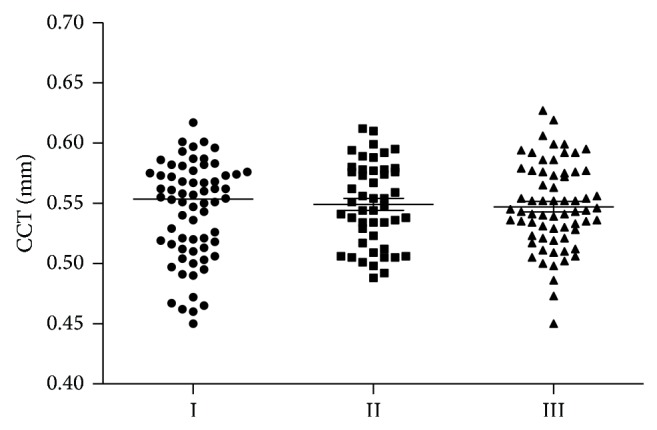
CCT (in mm) values in patients with JOAG (I group), OH (II group), and the control group (III group).

Median value for CCT in patients with JOAG was 0.554 mm and was not significantly different compared with the control group (0.544 mm). Median value for central corneal thickness in patients with ocular hypertension was 0.55 mm and was not significantly different compared with the control group. There were no statistically significant differences between median values for CCT of I–III groups (*P* = 0.88; Kruskal-Wallis test). CCT values in three examined groups are presented in Figure 2. ECD and CCT values in three examined groups are also presented in Table 1.

There were no statistically significant differences between mean ECD (*P* = 0.224; Kruskal-Wallis test) and CCT (*P* = 0.775; Kruskal-Wallis test) for 1–4 groups, treated with different types of topical antiglaucoma drops. Median values for ECD and CCT in these four groups are presented in Table 2.

## 4. Discussion

Evaluation of corneal endothelium in children and adolescents can be especially important, whereas a higher rate of endothelial cell loss at relatively young age may have negative long-term consequences for vision in future life. The mechanisms leading to lower cell counts in patients with glaucoma are not clear. Gangon et al. formulated three hypotheses: (1) damage from direct compression of the corneal endothelium due to higher intraocular pressure, (2) congenital alteration of both the corneal endothelial cell layer and the trabecular meshwork in patients with glaucoma, and (3) glaucoma medication toxicity [11]. They observed a reduction of 13.0% in corneal endothelial cell density in patients with primary open-angle glaucoma and 11.9% reduction in normal-tension glaucoma patients. These observations were also confirmed by Cho et al., who reported that adult patients with primary open-angle glaucoma had significantly lower endothelial cell counts (2370.5 cell/mm^2^, *P* < 0.001) than the normal group (2723.6 cell/mm^2^), but there was no significant decrease in corneal endothelial cell density in eyes with normal-tension glaucoma [7]. They concluded that elevated intraocular pressure likely affected the decrease of ECD in eyes with glaucoma. In the current study median value for ECD in adolescents with JOAG was 2639.5 cells/mm^2^ and was significantly lower than in the control group (2955.5 cell/mm^2^, *P* < 0.0001). Similar results were obtained by Wenzel et al., who noted that the ECD was found to be 2780 cell/mm^2^ in congenital and juvenile glaucoma [12]. Lower endothelial cell counts were also proved by Żarnowski et al., who reported that ECD = 2337 cells/mm^2^ in patients with juvenile glaucoma [10]. Guigou et al. measured ECD in 69 glaucoma eyes of pediatric patients between 3 and 18 years of age [13]. The mean endothelial cell density in glaucoma eyes was 2922 cell/mm^2^ and it was significantly lower than that in the control group (3470 cell/mm^2^). Melamed et al. in experimentally induced intraocular pressure elevation in rabbit observed morphologic changes in corneal endothelium, which are associated with decreased corneal endothelial density [14]. They proved that high IOP may impact on the cornea in two ways: (1) elevated IOP influences the metabolic active-pumping mechanism, thus reducing resistance to aqueous flow to the stroma and consequent stromal edema, and (2) high IOP causes morphological cellular damage, for example, cellular ruptures, swelling of mitochondria, disorganisation of endoplasmic reticulum, and the existence of myelin bodies. Setälä suggested that high IOP and long duration of elevated IOP before glaucoma treatment may affect the endothelium directly or may cause hypoxic damage indirectly [15]. Unfortunately, we did not know the values of ECD and CCT in our patients with juvenile glaucoma before starting antiglaucoma drugs treatment, so we do not know about the influence of elevated IOP before starting medical treatment. To avoid the situation, when any changes in topical antiglaucoma drops were necessary due to IOP rises in JOAG adolescents, we decided to exclude such patients from the study.

There were no significant differences in CCT among three examined groups. All examined eyes had no biomicroscopic signs of visible corneal edema. In our study CCT was 0.554 mm in JOAG patients, 0.55 mm in OH patients, and 0.544 mm in control group, so the mean value of CCT of eyes with JOAG was thicker than that of normal eyes by 0.01 mm (10 *μ*m) but this difference was not significant. Lopez et al. examined CCT in a large group of 141 pediatric eyes with glaucoma (mean 0.598 mm) and 76 pediatric eyes at risk for glaucoma (mean 0.604 mm); CCT in 66 normal eyes was 0.558 mm [16]. They observed that mean CCT values in children with glaucoma extend far beyond values reported for normal eyes, so they suggest caution in application of standard formulas for IOP-to-CCT correction in these patients. On the other hand, Tai et al. who examined central corneal thickness in patients with childhood glaucoma observed that patient with pediatric glaucoma and a larger corneal diameter was more likely to have thinner CCT [17].

Many studies have been conducted to evaluate the effect of different antiglaucoma topical medications on CCT and ECD in adult patients [6, 7, 18–22]. We tried to determine whether the use of these kinds of drugs influences corneal endothelium and central corneal thickness in adolescents with juvenile open-angle glaucoma. Bourne and McLaren observed that among the available topical drugs, only dorzolamide seems to have a possible negative effect on the corneal endothelium [23]. In the study of Lass et al. timolol, betaxolol, and dorzolamide were found to be equivalent in terms of corneal endothelial cell loss and thickness after 1 year of therapy in 298 adult subjects with ocular hypertension and open-angle glaucoma with normal corneas [6]. In another study the mean ECD change in three groups of 369 adult patients treated with latanoprost, fixed combination latanoprost-timolol, and timolol was 0.3 ± 2.2%, 0.1 ± 1.8%, and 0.0 ± 2.5%, respectively, and was comparable with the rate of change of approximately 0.6% cell loss annually over a 10-year period of time in a normally aging population [18]. In our patients with JOAG we used topical carbonic anhydrase inhibitors, prostaglandin analogs, carbonic anhydrase inhibitor/beta-blocker combination (which all are licensed in children with glaucoma in Poland), and cardioselective beta-blocker, which was used in an off-label manner, because it is not licensed for paediatric use in our country. Furthermore, many glaucoma medications commonly used in children still do not have paediatric dosing and safety labelling information in any country. No significant complications were found regarding topical treatment, which is consistent with previous reports [24, 25]. We did not observe significant changes in corneal thickness or in endothelial cell density in the small sample of our patients who were examined after treatment with different kinds of antiglaucoma drops. Topical dorzolamide appears to be well tolerated and IOP reduction of up to 23% in children younger than 6 years [26]. Topical carbonic anhydrase inhibitors reduce the corneal pumping function, resulting in increased corneal water content and corneal thickness [27]. However, this side effect appeared only in patients with compromised corneas and a previous history of corneal pathology. Dorzolamide is clinically effective when used alone or in combination with other topical antiglaucomatous medications [24]. In study of Inoue et al. the endothelial cell density did not change significantly after topical 1% dorzolamide treatment for 3 months, and CCT was significantly increased [19]. Kaminski et al. reported that in patients treated with 2% dorzolamide mean corneal thickness was slightly increased on day 1 and returned to baseline measurements at the following visits, and endothelial cell count showed no change [27]. Giasson et al. tried to investigate whether dorzolamide alters corneal hydration control in patients with glaucoma or ocular hypertension [20]. They did not observe significant changes in corneal thickness or in endothelial cell density in the small (17) sample of patients who were examined. They concluded that in patients with glaucoma or ocular hypertension with normal endothelium and without baseline corneal edema inhibition of carbonic anhydrase with dorzolamide does not seem to affect corneal hydration control. The results of many studies suggest that dorzolamide and preservative-free dorzolamide are not significantly toxic to corneal endothelium under usual ocular conditions [18–20, 28]. It is in agreement with our results: ECD and CCT values in eyes treated with dorzolamide were similar to values of ECD and CCT in eyes treated with other antiglaucoma eye drops (Table 2). Another topical antiglaucomatous medication used in pediatric glaucoma is prostaglandin analogs. Prostaglandin analogs have become a favorite among practitioners because of their superior efficacy and an excellent safety profile. Latanoprost has been proven to be a safe and effective agent for lowering IOP in the management of pediatric and juvenile glaucoma and ocular hypertension [29, 30]. Ayaki et al. reported that latanoprost and travoprost did not exhibit lower cell viability to human corneal endothelial cells than BAK alone [28]. In current study 22 eyes were treated with latanoprost and their endothelium cell density was similar to eyes treated with other kinds of antiglaucoma eye drops.

Several studies have been conducted to evaluate the effect of antiglaucoma medications on CCT. In the current study we demonstrated that there were also no significant differences in CCT among the eyes treated with different topical antiglaucoma medications. Similar results were obtained by Wierzbowska et al., who observed that CCT appears not to differ in eyes treated with different type of topical antiglaucoma drugs either in monotherapy or combined therapy [22]. Different observations were done by Stefan et al. and Viestenz et al., who have shown that topical prostaglandin analogues onto the cornea reduces the CCT [31, 32]. The authors attributed these changes to upregulation of matrix metalloproteinases and subsequent effects on the extracellular matrix of the corneal stroma [32]. Viswanathan et al. also reported that CCT fell significantly in 187 eyes treated with topical antiglaucoma medications for at least 3 years: mean CCT reduction was 12.29 *μ*m [33]. Among treated eyes, CCT reduction was significant in those treated with either prostaglandins or a combination of prostaglandin and beta-blockers, while no significant reduction occurred in eyes treated with only beta-blockers when compared with control eyes [33]. On the contrary, there is evidence that topical use of CAI and timolol leads to significant increase in CCT [21, 32]. Lass et al. reported the changes in mean central corneal thickness of 1% or less in patients treated with latanoprost, fixed combination latanoprost-timolol, and timolol [18]. Korey et al. found no significant difference in ECD and CCT between patients with normal IOP, with untreated OH, with treated OH, and with primary open-angle glaucoma [34].

The majority of IOP-lowering drugs contain preservative, and long-term treatment with preservative-containing eye drops is known to cause ocular surface disease [35, 36]. Benzalkonium chloride (BAK) is the most popular preservative and it is considered to have harmful effect on the ocular surface. It is known that BAK is also toxic for corneal endothelial cells and several clinical cases have proved this fact [37, 38]. However, the toxicity of antiglaucoma drugs to corneal endothelial cells remains elusive. Gangon observed that those subjects receiving three or four glaucoma medications had lower cell counts than those receiving one or two medications [11]. Lee et al. showed that long-term treatment with BAK-containing antiglaucoma medication appears to be the main contributor to corneal toxicity [35]. Ayaki et al. evaluated the toxicity of antiglaucoma medications to corneal endothelial cells using an in vitro toxicity assay and they observed that it depends on the presence of BAK [28]. They concluded that corneal endothelium damage due to antiglaucoma eye drops may occur only in rare cases. Based on the existing data, it seems rational to support the use of benzalkonium-free solutions whenever possible, especially in young patients who expected to need multiple and prolonged topical treatments. It is unfortunate that these preservative-free antiglaucoma drugs are often not licensed in children.

Potential limitations of our study should be mentioned. The most important limiting factor is the fact that we did not know the values of ECD and CCT before starting antiglaucoma drugs treatment and their corneal endothelium might have been exposed to IOP rise before their first visit to ophthalmologist. Secondly, the rarity of juvenile glaucoma and ocular hypertension in this age group made it challenging to enroll patients. Consequently, statistical differences in ECD and CCT were difficult to determine given the small numbers of patients in this study. Third, the mean time of topical antiglaucoma drugs treatment is short. Undoubtedly, longitudinal studies involving large sample numbers of patients with JOAG are required.

In conclusion, our study confirmed that JOAG and OH did not affect CCT, but JOAG has influence on ECD in adolescents. There were no statistically significant differences between ECD and CCT of eyes treated with different kinds of topical antiglaucoma medications.

## Figures and Tables

**Table 1:** Median values for ECD and CCT with lower quartile (Q_1_) and upper quartile (Q_3_) in three examined groups.

	Group I with JOAG *n* = 66 eyes Q_1_; Me; Q_3_	Group II with OH *n* = 48 eyes Q_1_; Me; Q_3_	Group III Control *n* = 66 eyes Q_1_; Me; Q_3_
ECD	2445; 2639.5; 2937	2705.5; 2924.5; 3059	2813; 2955.5; 3043
CCT	0.513; 0.554; 0.574	0.52; 0.55; 0.577	0.523; 0.544; 0.576

**Table 2:** ECD and CCT in four groups treated with different classes of topical antiglaucoma medications (CAI: carbonic anhydrase inhibitors, PGA: prostaglandin analogs, BB: beta-blocker, and CAI-BB: carbonic anhydrase inhibitor/beta-blocker combination).

Antiglaucoma drug	*n* (eyes)	ECD Q_1_; Me; Q_3_	CCT Q_1_; Me; Q_3_
(1) CAI	18	2344; 2730; 3068	0.519; 0.556; 0.576
(2) PGA	22	2599; 2773.5; 2972	0.526; 0.558; 0.568
(3) BB	20	2331; 2539.5; 2782.5	0.493; 0.532; 0.578
(4) CAI-BB	6	2445; 2551; 3546	0.52; 0.544; 0.574
		*P* = 0.224^*^	*P* = 0.775^*^

^*^Kruskall-Wallis test.
